# Living on social assistance with chronic illness: Buffering and undermining features to well-being

**DOI:** 10.1186/1471-2458-10-754

**Published:** 2010-12-06

**Authors:** Anneli Marttila, Eva Johansson, Margaret Whitehead, Bo Burström

**Affiliations:** 1 Division of Social Medicine, Department of Public Health Sciences, Karolinska Institutet, Stockholm, Sweden; 2 Division of Global Health/IHCAR, Department of Public Health Sciences, Karolinska Institutet, Stockholm, Sweden and Nordic School of Public Health, Gothenburg, Sweden; 3 Division of Public Health, School of Population, Community and Behavioural Sciences, University of Liverpool, UK

## Abstract

**Background:**

In Sweden, the social security and sickness insurance systems are comprehensive and aim to provide people whose illness prevents them from earning their own living, with either sickness benefits or disability pension. Some, however, are not entitled to these benefits or receive social insurance benefits at a level too low for subsistence, and are referred to social assistance. The purpose of this study was to explore in depth how social assistance recipients with chronic illness perceive and respond to the experience of living on social assistance.

**Methods:**

Seventeen in-depth interviews were carried out with chronically ill people who had received social assistance for several years. Grounded theory informed the design of the study.

**Results:**

The study showed that different strategies (living one day at a time, taking steps forwards and backwards and making attempts to find ways out of the situation) were employed by social assistance recipients to maintain or improve their well-being. Contextual features like the prevailing welfare system, public services and the local neighbourhood could buffer or undermine these strategies and their overall well-being. These features together influenced how interviewees perceived their situation, the possible ways out of the situation and the consequences for their well-being.

**Conclusion:**

From this study it is evident that the way in which individuals on social assistance interact with services and how they are treated by professionals plays an important role in their well-being, in combination with what kind of help and support is available for recipients through the welfare system. In this respect, persons living on social assistance with chronic illness are particularly vulnerable. This study suggests that more effort should be made to find long term solutions concerning income support, rehabilitation and other services provided to this group.

## Background

In Sweden, the social security and sickness insurance systems are comprehensive and aim to provide people whose illness prevents them from earning their own living with sufficient income, either through sickness benefits or through disability pension, based on the principle of income replacement [1]. Nevertheless, as the system is designed to cater for those who have worked for some time, there is a proportion of the population who do not qualify for such benefits. Their last resort is social assistance, provided by the municipal social services. Young people, single mothers and people with an immigrant background are common groups among recipients of social assistance. [2,3]

Social assistance is a selective, means-tested benefit aiming to guarantee a reasonable standard of living. Local governments administer and finance social assistance and work under a national legal framework [2,4]. In 2009, about 237 000 households in Sweden received social assistance on at least one occasion during the year (5.8% of the population). Social assistance is considered to be a temporary resort but in 2009 over one third of all recipients were classified as long-term recipients (had received social assistance for at least 10 months). [5]

In our recent study among social assistance recipients [6], a substantial proportion reported chronic illness. People who are ill and seeking social assistance may be categorised in two groups: those who have a medical certificate showing that they are not able to work, but whose sickness insurance or disability pension is not sufficient or who are not entitled to other sickness benefits; and those whom social welfare professionals judge incapable of work at the moment because of social or medical reasons (for example drug or alcohol abuse, mental ill-health, neuropsychiatric problems or musculoskeletal pain). It is difficult to estimate the proportion of recipients of social assistance who are chronically ill. In their statistics, municipalities have different ways to code reasons for receiving social assistance and there are many different codes which are also health related (not all clients disclose that they have health problems). For instance, in a study in Stockholm 2007 [7] 37 percent of all adult recipients of social assistance were classified as being ill (including both mental and physical illnesses, and drug related ill-health).

The experience of receiving social assistance may differ for individuals, and may vary depending on the reasons for not being able to support oneself. Some clients in society are considered as more deserving than others. Van Oorschot talks about "deservingness rank order in European welfare states", that people tend to support the provision of welfare for elderly and sick people and those with disabilities more strongly than welfare for unemployed people or immigrants [8]. Recipients of social assistance are understood as the "least deserving" in this deservingness rank order. The set-up of a society, including social security systems and levels of benefits, and the views of the public on recipients of social assistance all colour the experience of being a social assistance recipient, which varies between societies [8-11]. In addition, the individual's own view of deserving or not deserving the assistance of society may influence their experience of living on social assistance. Besides having a limited income, living on social assistance may also have other meanings for recipients, for example loss of autonomy and being dependent on social services, feeling shame and not be part of the rest of the society [6,9,12,13], which may affect health and well-being.

According to Salonen (1993) needs-based assistance is always subject to debate and also follows business cycles. Means tested social assistance has low legitimacy in the general population. In times of recession there is more questioning of the system and groups receiving assistance. [10] Social assistance recipients are stigmatised by labelling, to separate them from other groups receiving other forms of state assistance. At the same time attention is shifted from societal causes to unwanted or deviant behaviours and individualization of the causes of welfare recipiency. The public discourse regarding needs-based assistance is thereby stigmatising, and the recipient perceives shame and being excluded. [10,14]

In our previous study among social assistance recipients [6], chronic illness was a common feature, but we found few other studies concerning those who were chronically ill and receiving social assistance in Sweden, and how this was perceived by the recipients themselves. From a public health and policy perspective it is important to study this group, as they are disadvantaged not only in terms of having a chronic illness, but also in terms of being dependent on social assistance for their subsistence which may further exacerbate their ill health and contribute to social exclusion. The purpose of this study was to explore in depth how social assistance recipients with chronic illness perceive and respond to the experience of living on social assistance.

## Methods

The study reported here is part of a larger study in Stockholm aiming to study experiences of living on social assistance in Sweden [6,15], and also a part of a comparative project between Britain and Sweden, which aimed to identify and compare the influence of policies and services on resilience in poor households. This comparative project was one component of the ESRC Human Capability and Resilience Network, concentrating on resilience in the face of socio-economic disadvantage and poverty [16].

In our previous study among 33 social assistance recipients [6], two overarching themes emerged: material and psychosocial dimensions of living on social assistance. Interviewees' explanations of what led them to claim social assistance were also described. Seventeen of the 33 interviewees stated that they had had work incapacity due to illness or disability for more than one year. [6] Therefore, we decided to investigate further how these interviewees perceived their current situation when living on social assistance. Usually the concept of illness refers to the subjective response to feeling unwell [17]. In this study we rely on self-reports of interviewees. The guiding methodological approach was grounded theory [18]. Grounded theory was regarded as suitable as the study was explorative and aimed to investigate the perceptions of living on social assistance and social processes shaping these perceptions.

### Setting and sample selection

We conducted fieldwork in six sites in Stockholm County, providing a variety of geographical settings with differing socioeconomic compositions. According to the Regional Ethical Review Board in Stockholm, section 5, the study did not require ethical approval under local guidelines (dnr 04-609/5).

We employed a purposive sampling strategy [19,20], which generates a sample suited to the specific needs of the study and the research questions. We did not decide in advance the number of interviewees or the type of categories they would represent; the number was determined during the sampling and initial analysis of the data. Our primary purpose was to explore experiences of people living on social assistance. The secondary purpose was to gather data from people with potentially different experiences (both positive and negative) to enable to contrast their experiences of living on social assistance.

The inclusion criteria for sample selection were that a participant should be of working age (between 18 and 64 years), currently receiving social assistance as their main source of income or as a supplement to their other incomes and willing to be interviewed. We aimed to recruit a heterogeneous sample of social assistance recipients comprising both men and women in different ages, including for example long-term unemployed people, lone mothers, and immigrants to Sweden. All these groups are common among social assistance recipients in Sweden [2,3,10].

To recruit interviewees, we first contacted units working with social assistance in each of the study sites and came into contact with some of those interviewed through social workers. We then contacted other places to which social assistance recipients were often referred, including several centres for labour market activities for people receiving social assistance. Most of the individuals asked to participate agreed. Three of those who were recruited did not attend arranged interviews for different reasons. Several interviewees did not attend the first time we had booked our interview. We arranged a new time and in some cases a third time. Hence, the recruitment was time consuming. We ended the data collection when we felt the "saturation point" was reached, that more interviews would add so little information that it would not be meaningful to conduct further interviews [20].

The first author (AM) conducted 33 in-depth interviews. The length of the interviews varied from 40 minutes to two and a half hours. Most interviews were carried out in Swedish, but one in Finnish and one in English. A further three interviews were conducted with the aid of an Arabic speaking interpreter, when the interviewees felt they could not express themselves properly using Swedish. Before the interview, the aim of the study was explained to the interviewees, who were informed of their right to withdraw and that responses would be kept anonymous.

An interview guide was used including open-ended questions about the daily life of interviewees, their contacts with services in the community, what made them feel well and what bothered them most, and how they managed their finances. Interviewees were also asked to reflect on how they felt about their health and the future. The interviews took place at social welfare offices, at the local public library or in activity centres to which social assistance recipients were referred. Interviews were tape recorded with permission, and were conducted in 2005-06.

The present analysis focuses on how those 17 interviewees who reported having chronic illness perceived their current life situation when living on social assistance. They were selected for this analysis and are the subjects of this paper.

### Study participants

Demographic characteristics of the interviewees are presented in Table 1. Twelve of them had children.

**Table 1:** Demographic characteristics of the interviewees

Characteristics	No of interviewees (17)
*Gender*	
Female	11
Male	6
*Age*	
19-30	4
31-49	9
50-63	4
*Ethnic background*	
Swedish	5
Second generation immigrant	1
Foreign born	11
*Marital status*	
Single	5
Married/lives with a partner	4
Divorced	7
Widow	1
*Educational background*	
Academic	1
Upper secondary school	3
Basic compulsory school or secondary school	10
No diploma/not completed school	3

All 17 interviewees reported that they had an illness which had lasted for more than a year. Interviewees were a heterogeneous group of people concerning their ill health and troubles they had with their illness. Both physical and mental illnesses were reported (e g epilepsy, heart disease, musculoskeletal pain, depression, anxiety and panic attacks) as well as drug/alcohol related health problems. Some interviewees had experienced times in their lives when they could not even get out of bed and manage daily routines like shopping and cooking. Some interviewees had quit taking drugs and alcohol and suffered from health problems related to that. Some had mental health problems, e g depression and panics attacks, and were in the middle of the recovery process to manage their daily lives with their illness.

Fourteen interviewees had social assistance as their main source of income. Ten interviewees had lived on social assistance for more than five years; four interviewees reported that they had received social assistance from time to time over several years. At the time of the interview three of the interviewees had disability pension, at a level insufficient for their subsistence, which meant that they could qualify for social assistance as a supplement to their pension. In such a case social welfare offices did not make specific demands on them. Some of the interviewees said they participated in labour market activity, several that they were waiting for an examination of their work capacity, and some said they were not participating in any such activity at the moment because of their health condition.

### Analysis

All interviews were transcribed verbatim and were analysed initially with open coding, whereby the researcher assigns codes to pieces of text, going through each transcript, line by line [18]. The paradigm model was a guiding principle in axial coding where data is put back together in new ways by making connections between categories and subcategories [18]. For example, we identified interviewees' strategies to manage their daily life as an emerging category. In the final stages of the analysis the core category of the study was developed and selected (selective coding) and through this we made sense of the relationships between categories and subcategories developed during the analysis. The Atlas.ti 5.6.2 software (Scientific Software Development, Berlin) aided this process.

Data analysis followed the constant comparative method [18,21] through ongoing comparisons of categories from one case to the next (for example experiences, situations, actions), within a case and comparing incidents with incidents. All the authors were involved in discussions about emerging categories and the analysis of the data. The names of interviewees in the accounts in the results-section have been changed to protect interviewees' anonymity. Quotations were selected to illustrate the issues that emerged from the data.

## Results

The findings fell into four main categories. Three categories concerned how interviewees responded to their daily life. Different strategies (living one day at a time, taking steps forwards and backwards and making attempts to find ways out of the situation) were identified in the accounts as ways that the individuals managed the situation and maintained or improved well-being. The fourth category was labelled as the core category of the study and identified those features found to buffer or undermine interviewees' well-being.

The categories found in the analysis were seldom conscious choices or something the interviewees planned, but we could identify them in the accounts they gave. The identified categories often occurred simultaneously, and were not mutually exclusive. How interviewees perceived their situation depended also on other circumstances (e.g. experiences of labour market practice, family members' health situation) during the period being ill and living on social assistance.

**Figure 1:**
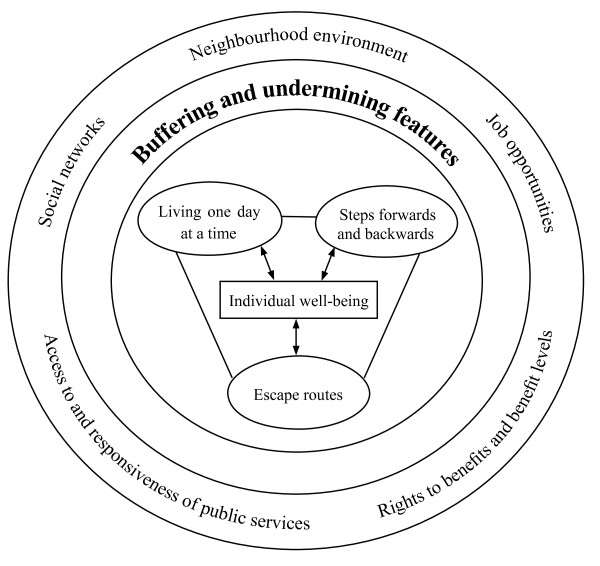
**Conceptual model of buffering and undermining features influencing well-being**. At the centre of the model is the individual well-being for social assistance recipients who are chronically ill. The three categories immediately surrounding that (living one day at a time; steps forwards and backwards; escape routes) all influence well-being and interact with one another. Contextual features like the welfare system including rights to benefits and benefit levels, access to and responsiveness of services as well as social relations and networks could buffer or undermine individuals' strategies and their well-being as a whole.

Figure 1 was constructed to illustrate the conceptual model emerging from the data about influencing features and how they interact.

At the centre of the model is the individual well-being for social assistance recipients who are chronically ill. The three categories immediately surrounding that all influence well-being and interact with one another. The interviewees had a short time perspective in their lives. They did not plan the future because the future was so uncertain and they perceived their opportunities to influence it as limited. Living one day at a time or "today" was their way to lessen the stress over the economic, health-related and social situation. In this sense the short time perspective acted as a buffer in this situation. At the same time living this day was also undermining as they had no opportunity to make plans for the next week or month, or the future.

The process of improving health or life situation included both steps forwards and backwards for most of the interviewees. For some the change to better health or social situation was rapid but for most it took time. Different small steps forwards, such as managing daily routines like shopping and cooking, were often necessary for major transitions like finding a job.

In the accounts we also identified interviewees' conscious or unconscious attempts to find "escape routes", ways out of the uncertain situation. Getting other benefits, like sickness benefit or getting a diagnosis were identified as such.

Contextual features like the prevailing welfare system including rights to benefits and benefit levels, access to and responsiveness of services as well as social relations and networks could buffer or undermine individuals' strategies and their well-being as a whole. These features were interwoven in interviewees' accounts.

### Core category: buffering and undermining features to well-being

The core category of the study is called "buffering and undermining features to well-being". These features were discussed directly in interviews or indirectly through talk about meeting professionals and being in contact with the welfare system. In Table 2 examples are given of these features which we have categorized at individual, neighbourhood, service and socio-political level, and which shaped the context in which interviewees perceived their situation when they lived on social assistance and had a chronic illness.

**Table 2:** Core category: Examples of buffering and undermining features to well-being identified in data at different levels

Levels	Buffering features	Undermining features
**Individual level**	Self-confident, social contacts, active orientation, feeling of being included in society	Destructive relations, traumatic life events, feeling hopelessness and frustration, isolation, health problems, family problems, feeling shame for receiving benefits
**Neighbourhood**	Feeling safe, good facilities in neighbourhood, access to nature and free time recreation	Poor access to local services, poor local communications, neighbourhood segregation, dirty and noisy local environment
**Services**	Provision of alternative activities, continuity in contacts, non-judgemental, being treated as an individual	Avoidance of services, bad experiences, making things worse, mistrust in communication, risk to get stuck in activities, short term perspective
**Socio-political level**	Right and access to benefits, adequate benefit levels, access to child care, good employment situation in the country	Low income, unemployment, immigrant background/new in Sweden

At the individual level, several features were discussed as buffering (for example feeling safe and having self-confidence) in hardship. Undermining features were related to the economic situation, social relations or psychological aspects (like feeling frustration and hopelessness). These features also influenced how the interviewees reacted in adversity. This can be illustrated for example by how Lisa, a 25 year old woman who suffered from panic attacks, responded when she was asked about what is most important in life:

*For me it is to feel safe, have a balance somehow in life between difficult things and good things. Right now I don't have that balance. But if you can find that feeling, which I think is possible, then you have an inner peace. And that, I think, is what you should have*.

Good relations with friends or children gave strength and quality of life in hardship for several interviewees. Good facilities in the neighbourhood and in the local community as well as access to and quality in public services and contacts with professionals were perceived as important. A key issue concerning services was how professionals treated their clients. Nina, a 43 year old woman, described her experiences of being in contact with health and social services after years of heavy drinking, destructive relations and traumatic life events. She perceived she had been treated as an individual, taken "seriously". She said:

*I don't feel that I am a piece of paper among other papers on their desk and that feels good. When they treat you as an individual, it is also easier to talk about my situation...And then it becomes easier for social services to understand which kind of needs I have. Their role as I see it, is to push us back to society...sometimes it takes a short time and sometimes a longer time. It depends; we all have different things to deal with*.

Access to nature and leisure time recreation were other examples of important buffering features especially in the situation with limited economic resources. On the other hand, where there were family problems or destructive relations; where access to services was poor or services were perceived as non-supportive; or where the immediate environment was poor, these features acted to undermine the well-being of individuals. One example of these undermining features was when the contacts with authorities made interviewees feel worse. As social assistance recipients they had to act as they were told; otherwise they did not get their benefits. They had to answer to a lot of questions about their private life. Some interviewees described they felt "*as a little child*" when being in contact with social welfare.

At the socio-political level, the accounts referred to the context in which social and health services were implemented and to which rights, benefits and social and health services interviewees had access. Features such as rights and access to child care and welfare benefits, provided the basic opportunities for welfare and prerequisites to build on in the process of change. Most interviewees, especially those with an immigrant background, were worried about their own possibilities to find a job and earn a living, but were happy for the opportunities their children had in society. Ali, a 53 year old man with an eye-disease waiting for an operation had lived in Sweden for six years, and reported that all his four children had learned Swedish quickly through the day care and school. The oldest ones wanted to study medicine and social sciences at the university. Although they lived in such an insecure economic situation, Ali perceived that his children had opportunities for education and "*a better life*". He said: "*The future is theirs, not mine..."*

In the following section we present the three categories of strategies identified in the accounts as ways that individuals managed their situation and maintained or improved well-being.

### Living one day at a time

We found the category, "live life one day at a time", as a response to difficult circumstances; not thinking too much about the future and problems in life. To live on social assistance was in itself a strain; being ill at the same time increased the vulnerability. A way to lessen the stress over the economic, social and health situation was to live "today". This category includes two subcategories (uncertainty and shame with social assistance and between hope and despair).

#### Uncertainty and shame with social assistance

Living on social assistance was something most interviewees did not want. When the situation was not temporary, but long lasting, it meant not being able to live an independent life, to have to declare personal things to social welfare professionals, being dependent. Petra, a 27 year old woman with musculoskeletal pain was anxious about her situation:

*P: This life I live now, I don't want to have it. This is a total disaster*.

I: What in your life is a disaster?

P: I like to work, have something to do. But when I have this pain, it doesn't work. There are no options; I do labour market practice now, but for how long? Who knows? One month perhaps?

Economic insecurity was described as a stressful factor in everyday life. Interviewees did not know exactly when they would receive the payment from social services. Especially at the end of the month they used to worry. Lisa, a 25 year old woman who suffered from panic attacks described:

*Last days of the month, you live with the coins...It should be the 27^th ^we get the money, but sometimes things happen on the way. Your application is somehow missing or they have done something wrong and they tell you it is going to take three more days to fix. And the bills should be paid at that time and then I get stressed. You have to phone to the Electricity Company and your landlord and all the troubles that come with it..*.

The majority of the interviewees did not qualify for sickness benefit. Social assistance provided them with the opportunity to get by during the time they were sick and not able to work. However, they had to renew their application for social assistance each month and go through the same appraisal procedure over and over again. To apply for money every month was perceived negatively by most, especially when they were ill and health improvement was their primary objective.

Frustration over the situation was expressed in all interviews. Several of the interviewees also expressed shame over having to live on social assistance, even if it was not "*their fault*"; they were unable to work because of their poor health, labour market situation or family problems. There were several examples in the accounts of how shame was manifested in daily life, for example not telling the truth to other people when they asked what they did for their living. Annica, a 47-year old divorced woman with musculoskeletal pain, a mother of three children recounted her experiences when her vacuum cleaner broke down and she had to ask for extras from social welfare:

*It was very embarrassing. They have to come and see. They asked a lot of questions, how long I had that Hoover and so on. But I don't ask for a Hoover from social welfare if I don't need that. It really was a last way out for me. I asked my family, my relatives if they had any. They didn't have, otherwise I would never have asked social welfare*.

#### Between hope and despair

Some younger interviewees were hopeful and saw some chances to get better and find a job some day. Several interviewees in their 50 s were more hesitant because of their age and poor health. Ibrahim, a 48 year old man with a lung disease and pain in his leg, father of two small children said:

*I really want to work and provide for my family. But sometimes I think it is too late, I am too old...I am like half a person [with his health problems] but still I am trying*.

Several expressed frustration over their "*hopeless*" situation, especially those who had a low disability pension and supplemented their income with social assistance. They were ill, retired with disability pension and could not work. At the same time they constantly had problems with their household finances. Even the younger ones reported that they could not plan for their future, and hoped there would not be any unexpected expenses. Minna, a 29-year old woman, a mother of four children who had suffered from depression after domestic violence explained that she was "*successful*" in handling money, but the situation still was difficult:

*I don't dare to plan any future. Anything can happen on the way. So I take a day at a time. I don't even plan next weekend. I am not that kind of person who is planning because you never know. Hopefully it is going to get better some day. That is my goal. To find a job and get somewhere*.

### Two steps forwards, one step backwards

The category "two steps forwards, one step backwards", illuminates the process of being ill when living on social assistance, and trying to find solutions related to social, material and health issues. Sometimes the process consists of many steps forwards, but often also some steps backwards and then some forwards again. This category includes two subcategories (the process of change, and finding meaning in life or giving up).

#### The process of change

In interviewees' accounts, improvements in health or life situation came about through a process of small changes in daily routine so as to manage activities in everyday life like doing shopping, being able to go out for a walk, meeting a friend or getting help with something in daily routines. Some interviewees had also participated in labour market activities which in some cases the participants had perceived as not helping them that much. In other cases the impact of the labour market activities was the opposite, they felt stronger after participating. For example Lisa, a 25 year old woman who suffered from panic attacks said that activities had helped her "*to grow*" and "*to feel safer*".

There were descriptions about both steps forwards and backwards. Nina, a 43 year old woman described her attempts to find a way "*back*" after years of heavy drinking. She realised that she wanted *"so much" *and "*too quickly*". She tried to participate in work rehabilitation and at the same time participate in a rehabilitation program for her alcohol problems while working half time:

*I really did not manage all that, mentally or physically. I think after the rehabilitation program it would have been better if I had been on sick leave a couple of months because I was not ready. The point of the program is to reflect on things. I had awful pain all the time and at the same time a thousand things on my mind. I felt I was going crazy. I was so tired. One morning I woke up and I did not manage anymore*.

#### Finding meaning in life or giving up

Different small steps preceded major transitions like attempts to find a job or get a diagnosis of the health problem or get another source of income. Four subcategories could be identified influencing these steps forwards: finding meaning in life despite difficult circumstances; enjoy small things in everyday life like being able to go out and take a walk; children and social contacts giving joy (interaction with others); or interests giving joy.

Several interviewees described how they found meaning in life even when living in difficult circumstances. Having children "helped" them to see the positive aspects in life. Children were described as one of the most positive things in life, the adult children helping them and the joy they had to watch the younger ones grow up. Many interviewees were proud of their children and felt stronger when they talked about them. Marcus, a 45-year old man suffering from health problems after years of drug abuse said:

*My daughter is very important for me. I will see her growing older and all stuff with the family and so on. I want to see her happy and have a good life and if I can I want to help her as much as possible*.

Several interviewees who had adult children had assistance from them, both in social and material aspects. For example Leyla, a 42 year old divorced woman who had problems with restless legs and back pain, described how her son helped her every month to pay part of her rent because the social welfare office did not pay the whole amount (her flat was considered too big and expensive for her). Hossein, a 55 year old divorced man with disability pension, described how his son helped him with all kinds of daily routines like taking a shower and shopping food (he was not able to manage these things by himself because of his ill-health).

There were, however, also some interviewees who expressed problems with their family situation and relations. Some interviewees had children who were addicted to drugs, and some had children with learning disabilities or difficulties in school. Several interviewees also discussed domestic violence or partners who were drinking or taking drugs. Their difficult home situation did not help improve their health. In these cases the effect was the opposite; they felt worse. Susan in her 50 s told about her daughter's addiction to drugs and the chaos that brought in their life. She had financial troubles in her business for a period and ended up bankrupt:

*If I had felt well, I never would have ended up bankrupt. In that situation I had no strength to struggle. My daughter was taking drugs all the time and it was constantly something going on, problems in school. This had been going on for a longer time and I was ignoring difficulties with my income because I had no strength. We had no structure at all in our life at that time*.

Life was described in several interviews as quite isolated, because of their illness and material situation. Several interviewees said they could not afford to travel, take a vacation or have extras like going out to have dinner. There were however also those interviewees who could find joy in things which did not cost money: like taking a walk with their dogs or meeting their friends which also made them feel better. Ibrahim, a 48 year old man who was very frustrated over his situation liked the multicultural neighbourhood where he was living. The social contacts helped him to get some distance to his problems:

*...I think it is the contact between people here, the feeling of not being alone. You always have someone to talk to, you can sit on your balcony or in the kitchen, and someone is always passing by and saying hello, outside too when you are outdoors and playing with your children. I know most people here. I talk a little bit so you are active all the time. You don't have to sit alone with your feelings and think. I have relatives here, people from the same country as I am, other immigrants too...I can speak my language with them too. We meet and talk and do things together...If I lived in some of these affluent neighbourhoods I would not have this opportunity..*.

### Escape routes - external support

This category found in the accounts highlighted "ways out" and was related to the labour market or social and health services in some way or another. There were also a few interviewees who could not find any way out of their situation. One of them was Hossein, a 55-year old man with disability pension suffering from a lung disease and pain in his legs:

*I have so many problems with my money... When your income is limited, your life is limited...I am not able to see forward, I don't see any future...When you don't have any money, then the pain comes...If I don't have a good income, I can't have good health*.

Three "escape routes" were identified (job or labour market practice, having a good relationship with a professional and getting a diagnosis or other benefits).

#### Job or labour market practice

Working full time after a longer period of illness or unemployment was an option which was discussed with hesitation. Most interviewees hoped to "come back" step by step through part time work or some form of labour market practice. It was not easy to find a job after being outside the labour market for several years. Minna, a 29 year old woman with four small children was afraid to fail if she took a "too big step"; she wanted to find a part time work experience:

*...I have gone through so much; I am not able to seek a job. I can't get a permanent job because I am going to screw it up anyway. I have the strength to fight for two weeks and then I get tired. I have had so much trouble with things, private things, drug abuse [in her family] and this and that*.

#### Having a good relationship with a doctor or social worker

Professionals in both social and health services played an important role in interviewees' lives. They had the power to influence the help which interviewees could access. Meeting a professional who put their energy into trying to help was appreciated. In the accounts there were examples of these "good" meetings that acted as turning points in their situation. The change did not happen over a day but as a process with small steps forwards, which these contacts with professionals help made possible. One example of this was Marcus, a divorced man in his 40 s who explained his addiction and his struggle to quit. Having taken drugs for years, he decided to quit because he had no strength left to live that kind of life. He met a doctor who helped him to a get a chance to quit through access to treatment and he also met a social worker willing to support him. He explained important qualities of a professional:

*Most important is that you have a good contact, that you have trust in that person...I am glad that I got this girl [social worker], I have. She really has done more for me than she has to do. She really has helped me*.

#### Getting a diagnosis/getting other benefits

Getting sickness benefit was considered valuable as it indicated acceptance or confirmation of being sick, even if the payment was at a low level and needed to be supplemented by social assistance. For some it did not matter so much where they got the money from because the payment would be so low anyway. The most important factor was to get a diagnosis and know the cause of their illness. Petra, a 27 year old single woman with musculoskeletal pain was one of them:

*My pain is getting worse and worse because they have not done anything, not a single medical examination. They say I have problems with my muscles. But I have had this for over ten years now..*.

Annica, a 47-year old divorced woman with musculoskeletal pain, a mother of three children was waiting to do a work capacity appraisal. She got her income from social assistance instead of sickness benefit although she could not work because of her pain. She was not happy that social welfare professionals sent her to different activities which she felt were not suitable for her, with her health problems. She believed that if she got sickness benefit, they would probably have more understanding for her health situation.

As social assistance recipients, the interviewees did not automatically have access to rehabilitation. This depended on a range of factors, including the kind of health problem they were suffering from, in which municipality they lived and which professional they met.

Lisa, a 25 year old woman with panic attacks described how the professionals sent her back and forth between the unemployment office and the social welfare office because they thought it was unclear how much demands she was capable of managing. The unemployment office thought she could not manage full time work and the social welfare office judged differently. She explained further:

*All the time, I ran back and forth, back and forth between them. If I was not actively seeking jobs I didn't get any money. That is why some months I didn't get any money at all. And I had no energy to fight with them either...I suffered from panic attacks; I couldn't manage to take a bus or train because of that...and they said: seek jobs everywhere...It was impossible*.

## Discussion

Although the social security and sickness insurance systems are comprehensive in Sweden and aim to provide resources for people whose illness prevents them from earning their own living, there are people who are not entitled to these benefits and are referred to social assistance. This study illustrates the hardships associated with being chronically ill and living on social assistance in Sweden, and strategies employed by the individuals to manage the situation and maintain or improve their well-being. Furthermore, it describes how the interaction between individual and contextual features may buffer or undermine well-being. Living on social assistance is a strain, especially in the longer term, both for the individual and his/her family, and may also lead to social exclusion [13,14].

Social assistance clients in Sweden, including those with chronic illness and unable to work, have to renew their application for social assistance each month and go through the same appraisal procedure over and over again. Although not intended as such, for some individuals and groups social assistance has become a more or less permanent solution for their subsistence problems. Some interviewees indicated that they would prefer to have their income from social insurance, even if it were at a low level. With such an arrangement, at least there would not be the economic uncertainty associated with social assistance. Instead of income security, social assistance had become a source of permanent insecurity in their lives. This contributed further to worse health and well-being, as also indicated in other studies [7,9].

Arguably, chronically ill persons on social assistance have the same kind of needs for rehabilitation as those chronically ill persons who qualify for sickness benefit [22,23]. Several interviewees (for example those whose health problems started before they came to Sweden and those who had not yet entered the labour market) were not covered by services organised by the social insurance office and their possibilities for rehabilitation were limited. In such cases access to rehabilitation depended on the municipality in which interviewees lived, what kind of health problem they suffered from and which professional they met. Lack of rehabilitation and sometimes even lack of a diagnosis were hindering interviewees in the recovery process. Some interviewees, particularly those who had diffuse or undiagnosed health problems, perceived a lack of coordination between the different authorities involved. This is in line with findings of other investigations in Sweden [7,22], and suggests that other approaches need to be considered in order to meet the needs of chronically ill persons who do not qualify for the regular services.

Some interviewees expressed the view that it is "not their fault" that they had to rely on social assistance; in case of illness society should help and give support. As sick or disabled they saw themselves as "deserving" [8] which lessens the stigma associated with social assistance. In an earlier study Gunnarsson [24] found that younger and older women gave social assistance different meanings: for the younger social assistance was a way to be economically independent from their parents. The older ones, who in several cases were also ill, perceived social assistance as economic dependency and saw that finding a job or getting sickness benefit would be the solution. In our study, social assistance was not perceived in the long run as a route to independence, but as a source of dependency and insecurity, even among the younger. Social assistance still today is associated with guilt [25], shame and feeling stigmatised [9,10,12,14]. Several interviewees in this study as well as in our earlier study among social assistance recipients in Sweden [6] did not want to identify themselves as one of "those" who receive social assistance, but explained their situation as caused by external factors outside their reach, as poor health, family problems, poor labour market or discrimination.

Interviewees had contacts both with health care and social services, and were dependent on professionals and welfare benefits for their living. Services and professionals could be buffering or undermining depending on what kind of help and support interviewees were referred to or how interviewees perceived they were treated. There were several examples of how professionals and services had facilitated a positive change in the interviewees' lives. They felt they had been treated with respect and as individuals, without being judged. There were also examples of the opposite, when services and professionals "made things worse". Different professionals may also have qualitatively different views on clients, leading to "unequal encounters" and differences in opportunities for rehabilitation [26]. It is important that services and professionals especially consider the needs of individuals with chronic illness who depend on social assistance for their subsistence, as they may be in a particularly vulnerable and dependent position in relation to public services.

In an earlier study of ours [27] carried out in Britain among people living in poor households, positive changes in interviewees' lives depended on opportunities arising for them to release their potential in order to start further processes. The achievements in everyday life like being able to get children to school on time were examples of an "upwards moving spiral", small steps toward something more positive. In the present study, accounts also illustrated that the process of improving health or life situation included both steps forwards and backwards. Taking "too big steps" too quickly could result in regression and losing self-esteem.

Finding work or labour market practice was also found as an "escape route". The "dream" or desire was to "find work and get somewhere". However, most of the interviewees in this study were sceptical about finding and managing full-time job, with their health problems and having been outside the labour market for several years. A gradual and stepwise entry or comeback into the labour market would therefore be more realistic for many in this group.

The findings of this study suggest that health and social welfare professionals should focus on finding individually adapted solutions for their clients, as well as building trust and understanding about the strategies individuals use in adverse circumstances to maintain or improve their well-being. From a health and social policy perspective the links between ill health and social disadvantage should be recognised, and coordination between health and social services for this group needs to be improved. There is also a need to further develop ways of entering the labour market for social assistance clients with chronic illness. The maintenance of good quality in local health and social services benefits the population as whole, but especially the most vulnerable groups who depend on services for their living. Social assistance clients with chronic illness are a vulnerable group who risk "falling between the stools" in their contacts with health care providers and other welfare institutions.

### Limitations

This study was explorative and its results cannot be generalised. Nevertheless it has highlighted some experiences, strategies and needs of a particularly disadvantaged group in the Swedish society. In this study we did not present comparisons between interviewees living on social assistance with or without chronic illness. However, several categories (e.g. living one day at a time or having a good relationship with a professional) were found to be important among both groups in our data. Follow up studies of persons with and without chronic illness living on social assistance might shed further light on the development over time of these groups. Ethnic background was not a specific focus in this study but will be addressed in a coming study.

## Conclusion

Chronically ill persons living on social assistance face many adversities. How they perceive and respond to the experience of living on social assistance is coloured by different features at individual, neighbourhood, service and socio-political levels. Contextual features, including the way in which individuals interact with services, how they are treated by professionals and what kind of help and support is available through the welfare system, are crucial in this adverse situation. Policy making and public services should especially consider the needs of chronically ill persons living on social assistance, who may be particularly vulnerable to a further decline in their health and to social exclusion if they are in this situation for a prolonged period of time.

## Competing interests

The funding organisations had no role in the study design, data collection, analysis, interpretation or writing this article. The authors declare that they have no competing interests.

## Authors' contributions

AM participated in the design of the study, carried out the interviews, analysed the data and drafted the manuscript. EJ participated in the analysis of the data and preparing the manuscript. MW participated in the design of the study, in the analysis and preparing the manuscript. BB participated in the design of the study, in the analysis and preparing the manuscript. All authors had read, revised and approved the final version.

## Pre-publication history

The pre-publication history for this paper can be accessed here:

http://www.biomedcentral.com/1471-2458/10/754/prepub
